# Flowable Bulk-Fill Materials Compared to Nano Ceramic Composites for Class I Cavities Restorations in Primary Molars: A Two-Year Prospective Case-Control Study

**DOI:** 10.3390/dj7040094

**Published:** 2019-09-25

**Authors:** Maria Sarapultseva, Alexey Sarapultsev

**Affiliations:** 1 Department of Pediatric Dentistry, Medical Firm Vital EBB, 136 Shejnkmana str., 620144 Ekaterinburg, Russia; 2 Institute of Immunology and Physiology (IIP) of the Ural Division of Russian Academy of Sciences, 620002 Ekaterinburg, Russia; 3 Ural Federal University named after the first President of Russia B.N. Yeltsin, 19 Mira street, 620002 Ekaterinburg, Russian

**Keywords:** class I cavities, flowable bulk-fill materials, nano-ceramic composites, pediatric dentistry restorations, primary teeth caries

## Abstract

Background: The aim of this split-mouth study is to compare the results of 24 months’ clinical performance of primary molar Class I restorations with a nano-ceramic composite, *Ceram•X mono* (Dentsply) with a flowable bulk-fill material regular viscosity, *SDR* (Dentsply). Methods: Following the ethical approval, 27 patients with at least two class I cavities in primary molars were included in the study. A total number of 54 restorations were conducted (n = 27 for Ceram X and n = 27 for SDR). Restorations were evaluated at baseline, 6, 18, and 24 months, according to the modified Ryge criteria. The cavosurface marginal discoloration and color match were evaluated visually after air-drying the tooth and after removing the plaque (if necessary). Results: At 24 months’ follow-up, 54 restorations showed similar clinical performance. The statistical analysis did not reveal any statistical significance in the values between the groups in 7 out of 7 modified Ryge criteria. However, two restorations in both groups received Bravo ratings in the cavosurface marginal discoloration scoring. No side effects were reported by the participants of the study. Conclusion: Restorations with both materials (*Ceram•X mono* and *SDR*) have provided almost identical results.

## 1. Introduction

There is a continuing demand for the use of time-saving restorative materials in pediatric dentistry [1,2,3]. That is why one of the most common materials for the restoration of primary teeth are glass ionomer cements (GIC) [2,3]. However, despite the convenient handling properties, excellent biocompatibility and absence of necessity of adhesive preparation, the mechanical properties of GIC remain imperfect, and that is a critical factor for any long-term restorations [2,3]. Moreover, according to the literature, conventional GIC and resin-modified GIC materials are unsuitable for long-term restorations in high-stress situations [2,3]. This is why the bulk-fill resin-based composite (RBC) materials can probably be considered as a promising material for pediatric dentistry.

The bulk-fill RBC material class has been specifically implemented as an effort to speed-up the restoration process by skipping the time-consuming layering process, without adverse effects on polymerization shrinkage, cavity adaptation, or degree of conversion [4,5]. Bulk-fill composites are classified into high-viscosity and low-viscosity (flowable) types [6]. High-viscosity bulk-fill composites (such as *QuixFil*, Dentsply, Konstanz, Germany) can be used to fill cavities entirely with only one material up to the occlusal surface, whereas low-viscosity bulk-fill composites (such as *SureFil SDR flow*, *SDR*, Dentsply) equate a final capping layer by a regular hybrid composite material because of inferior mechanical properties towing to their reduced filler load and filler composition [7,8]. It was shown that bulk-fill RBCs had the lowest shrinkage stress and shrinkage-rate values in comparison to regular flowable and non-flowable nanohybrid and microhybrid methacrylate-based RBCs and a silorane-based microhybrid RBC and do not increase the intrapulpal temperature in primary teeth during the curing/setting [9,10,11,12]. They were also able to reduce cuspal deflection in standardized Class II cavities compared with a conventional RBC (*GrandioSO*, VOCO, Cuxhaven, Germany) restored in an oblique incremental filling technique [5,8]. Moreover, because of low-viscosity and easy handling properties of flowable bulk-fill RBCs, they are particularly beneficial in restoring cavities which are difficult to access and are particularly well-suited for patients with limited compliance, which is important when working with younger patients [13].

However, it should be noted that composites of low viscosity, like the so-called flowable bulk-fill composites, have certain technological features of restoration techniques. Thus, according to the manufacturer’s instructions, the bulk-fill material can be directly applied after cavity preparation, isolation, and proper treatment of tooth surfaces with adhesives. Replacing the occlusal 2 mm with any methacrylate-based composite as directed by the manufacturer should complete the restoration [7]. After the occlusal component has been sculpted, contoured, and cured, the restoration can be finished and polished in the preferred manner of the clinician. According to Ilie et al. (2013), the recommendation to finish a bulk-fill RBC restoration by adding a capping layer made of regular RBCs is an imperative necessity since the modulus of elasticity and hardness of bulk-fill RBC materials (*SureFil SDR flow, Venus Bulk Fill, and Filtek Bulk Fill*) are considerably below the mean values measured in regular nanohybrid and microhybrid RBCs [5]. The presence of the aforementioned additional stage, in the form of the application of the covering layer, puts the simplicity of the restoration technique, especially in pediatric dentistry, into consideration. 

However, the manufacturer’s instructions for some flowable bulk-fills indicate the possibility to use them for restoration of small cavities in primary teeth. Moreover, one case report described that the bulk-fill RBC was used in the primary dentition in the entire class II cavity without cover layer [14]. The results were quite satisfactory: the finished restoration showed a higher translucency, appearing slightly greyer. Thus, the authors proposed the possibility of approving the indication for entire restorations in the primary dentition because wear resistance and sculptability are not as important as in permanent teeth [14]. Later on, *SDR flow+* was approved by the manufacturer to be used in Class III and Class V restorations where a capping agent would not be used [15].

The aim of this split-mouth study was to evaluate the performance of primary molar class I cavities restorations with bulk-fill RBC *SDR* (Dentsply) compared with a nanoceramic composite *Ceram•X mono* (Dentsply) in a period of 24 months. The null hypothesis was that the above mentioned restorative techniques provide almost identical results.

## 2. Materials and Methods 

The study was carried out following the rules of the Declaration of Helsinki of 1975, revised in 2013. Ethical approval # C-11-07-2016 (11 July 2016) was obtained from the Institute of Immunology and Physiology of the Ural Division of Russian Academy of Science, Ekaterinburg, and informed consent was obtained from all parents or legal guardians of subjects recruited for the study. A research team representative approached families to ask if they would be prepared to have a research coordinator discuss the study with them. If yes, the coordinator discussed the research with the family (risks/benefits, voluntary participation, procedures). Families were given adequate time to reflect on the information, have any questions answered, and give free and voluntary consent. Patient consent forms (Supplementary Materials S1), incorporating Guidelines of Federal Compulsory Medical Insurance Fund of the Russian Federation (1999 # 5470/30-Z/I) and ADA Principles of Ethics and Code of Professional Conduct, were distributed to parents at the reception areas of dental clinics (ADA 2012).

Requested information included the name and date of birth of the pediatric patient, name, relationship to patient, and legal basis for adult to consent on behalf of the minor, description of specific treatment undertaken (in simple terms), alternatives to treatment, potential complications of the treatment, acknowledgement by the patient or parent/guardian that all questions were answered, and signatures of the dentist, parent or legal guardian, and witness. The report of the study was performed following the TREND guidelines (Supplementary Materials S2).

### 2.1. Sample Description

The sample size was estimated based on the assumed success rates of restorative materials [16,17,18] because no clinical trials were found to provide the success rate of primary molar restorations with flowable bulk-fill materials without capping. According to the performed calculations via OpenEpi software (Open Source Epidemiologic Statistics for Public Health, Version 3.01), 40 molars were required per restorative material group to detect and determine significant differences in outcomes at the 95% confidence level, with an alpha value = 0.05, and 80% power. However, according to Antczak-Bouckoms et al. (1990), split-mouth studies and cross-over designs required only one half the number of participants to produce the same accuracy as traditional, two-arm parallel clinical trials [19]. Thus, the theoretical sample size was set to 25 restorations per group and was increased to safeguard against possible drop outs.

### 2.2. Inclusion and Exclusion Criteria

The clinical inclusion criterion was the presence of at least two caries lesions with cavities of Class I on the second primary molars of the lower jaw. Caries lesions were diagnosed according to the recommendation of the European Academy of Paediatric Dentistry [20] and the American Academy of Pediatric Dentistry [21]. This study excluded patients older than 6 years old and those with a history of pre-excitation syndromes, motor impairments (cerebral palsy and epilepsy), and inadequate level of oral hygiene. 

**Figure 1:**
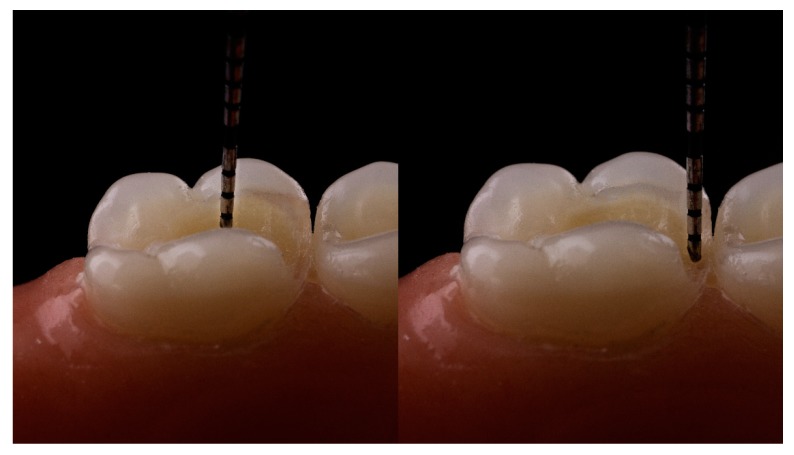
The distance between the cusp tip to pulp chamber ceiling, measured with a graduated probe.

The distance between the cusp tip to pulp chamber ceiling, measured with a graduated probe, as exemplified in Figure 1 ranged from 4 to 5 mm [13,22], while the thickness of 4 mm was indicated by the manufacturer as recommended for bulk-fill RBC materials application with one layer, and was proven to be cured “effectively” [23]. Thus, the cavity size of maximum 4 mm in depth and 3 mm in width was set as the limit to include in the study to avoid perforating the pulp chamber and to comply with the manufacturer’s requirements [15,22,23,24,25]. 

### 2.3. Study Population

The study was conducted in Vital EBB dental clinic in Ekaterinburg, Russian Federation. Total of 27 Caucasian children aged 3–6 years old (males 17 (63%), females 10 (37%)) were included in the study.

### 2.4. Study Interventions

After applying the inclusion and exclusion criteria, patients were selected for the study and were subjected to a thorough clinical examination. The research coordinator randomly assigned eligible patients to the two pediatric dentists. One operator did a dental exam and treatment on every single case. The examination required for each patient included the following: (1) medical and dental history, and (2) examination of maxillofacial area, oral cavity, dentition, and soft tissues. The two Class I restorations from different materials were placed in the oral cavity of each subject. One of the materials used (SDR), can only be used with a conventional viscosity composite coating with the manufacture’s exception of small cavities in primary teeth [15]. 

Cavities were prepared with turbine burs (NTI-Kahla GmbH Rotary Dental Instruments, Kahla, Germany) under water-cooling. The selective etching of enamel with 37% phosphoric acid and application of the bonding agent *Prime and bond NT* (Dentsply) was conducted after the disinfection of prepared cavities with 1% chlorhexidine. One cavity was restored with nano-ceramic composite *Ceram•X mono*. The second cavity was restored with flowable bulk-fill material *SDR* with normal viscosity composite. Occlusal adjustment was made with Arkansas abrasive stones (NTI-Kahla GmbH Rotary Dental Instruments), polishing with the *Enhance Finishing and PoGo Polishing systems* (Dentsply). 

The block scheme was used as a common randomization method for split-mouth study: Selection of initial side of treatment in the mouth and allocation of restorative material for each tooth were undertaken randomly using number sequences generated by MedCalc 16.8.4. Two lists of random numbers were created, one corresponding to the restorative material used (odd number for *Ceram•X™ mono* and even for *SDR*) and the other to determine the side of the mouth that was treated at that appointment, following the split-mouth design; at the other material on the opposite side was used. Each pair of numbers (from lists 1 and 2) corresponded to each patient. The operator has been blind to the random number schemes until just before placing the materials. The participants, independent observers, and the research coordinator were blind regarding the restorative material. Because *Ceram•X™ mono* and *SDR* each has recognizable characteristics, they were not blinded to the operator.

A total of 24 teeth with Class I cavities were treated with the above described technique and were evaluated three times during the 24-month period (at 6, 18, and 24 months excluding the initial evaluation at baseline). The observational period of 24 months was set as characterized by the minimum loss to follow-up [26] and regarding the expected longevity of primary tooth restorations [1]. Two independent observers evaluated the restorations according to the modified Ryge criteria (Table 1) [27]. The cavosurface marginal discoloration was evaluated visually after air-drying the tooth and after removing the plaque (if necessary). The same approach was used for color match evaluation.

## 3. Results

After 24 months, all 54 restorations (*Ceram•X mono* and *SDR*) were available for clinical evaluation in 27 patients (recall rate 100%). None of the restorations had shown any secondary caries or exhibited post-operative sensitivity at any evaluation point. The summary of clinical findings of modified Ryge criteria with respect to color match, marginal integrity, cavosurface marginal discoloration, and surface texture is shown in Table 2. 

**Figure 2:**
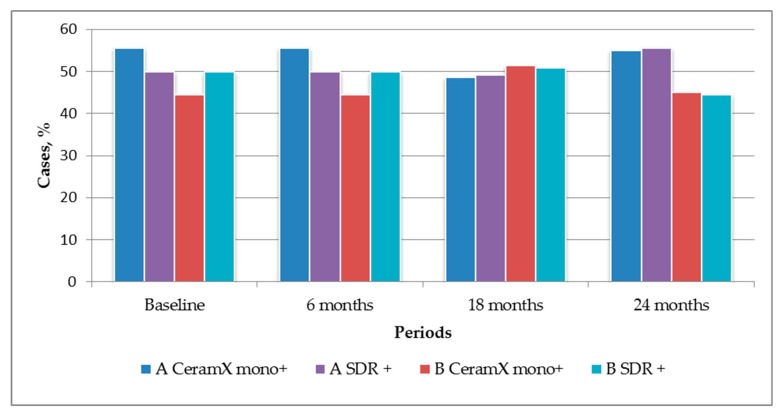
Percentage ratio of valuation amounts of two restoration materials (*Ceram•X mono* and *SDR*) during 24 follow-up.

The results of the statistical analysis (Table 3, Figure 2) confirmed that the differences in results between both treatment technique’s applications were not significant. The insignificant differences in column heights (Figure 2) indicate the similarity of the restorations scores of both the restorative materials (*Ceram•X mono* and *SDR*) used.

However, there was a difference in the marginal integrity at 24 months for criteria A and B (difference in cases = 1, difference in prevalence = 3.7%), presence and absence of secondary caries at 24 months (difference in cases = 1, difference in prevalence = 3.7%) and for A and B of surface texture between the restorative materials at 18 and 24 months (difference in cases = 1, difference in prevalence = 3.7%). Additionally, one restoration was revealed to be fractured at the time point of 24 months at *SDR* restoration group. For a deeper analysis, the comparisons of the same criteria among themselves for the two types of treatment were calculated. Deeper analysis was conducted using the timelines for both treatments. According to the results, the differences between the restorative materials (*Ceram•X mono* and *SDR*) at the end of 24 months were not statistically significant, and both materials used demonstrated the acceptable clinical performance.

## 4. Discussion

The group of bulk-fill RBC appears to satisfy the dental practitioner’s expectations, being more effective and less technique sensitive to place than conventional RBCs [24,28]. The implementation of these materials in dentistry practice has changed the application procedures, reduced the risk of entrapping air voids between subsequent increments with negative effects on mechanical strength, and, being time-saving, made the treatment procedure more economic [7,25,29]. 

With that, the growth of composite technology and regular introduction of new and “improved” versions of RBC materials have been so rapid in recent years, that long-term clinical data are rarely available [7,14,29,30]. One can find the interesting results of the longitudinal randomized controlled study investigated the performance of the posterior regular viscosity bulk-fill composite *QuiXfil*, but only one case report and one-year clinical study of Ehlers et al. (2013, 2019) have described the use of low viscosity bulk-fill RBC without cover layer in the primary dentition [7,14,30]. However, regarding the expected longevity of primary tooth restorations, even short with 1–2 year observational period studies, as a present one, can be of value to dentists, expand the range of knowledge, and set the directions for further long-term studies.

According to the results, all Class I cavities 54 restorations (*Ceram•X mono* and *SDR*) were available for clinical evaluation with both materials used having demonstrated the acceptable clinical performance. Class II and III cavities were not included in the study, as the manufacturer’s instructions postulated that non-composite SDR material with standard viscosity could only be used to restore Class I cavities [15]. This limitation allowed the exclusion of possible study errors related to the different distributions of the occlusal load on the teeth of the upper and lower jaws. The results obtained are consistent with the data of Ehlers et al. (2019) where no severe postoperative sensitivities or side-effects were reported and no statistically significant difference between the performance of flowable bulk-fill composite vs. a compomer was detected [30]. 

The conducted study confirmed the null hypothesis that the two restorative techniques employed provided almost identical results. However, practically similar results were obtained when applying these materials may have completely different significance if one looks at this issue through the eyes of a pediatric dentist. Working with children is quite difficult for a dentist. In contrast to adult dentist, pediatric dentist must take into account various factors which can influence the treatment such as age, cognitive development, pain perception, type of treatment, and so on [1,31]. All these variables play important roles in the selection and provision of dental treatment [32], but the main factor in the treatment of children is the time. In most cases, the children find it hard to tolerate the forced immobility, which may be accompanied by discomfort or pain. Thus, one of the main tasks of a dentist working with children is to minimize, but not at the expense of quality, the time needed for medical manipulations. Moreover, materials such as composite resins are highly sensitive to the technique and can be completely affected by the presence of water or saliva [33]. In children, where moisture and time control are critical, the correct restoration can be jeopardized and a low performance can be expected [33]. At the same time, the restoration technique with the use of flowable bulk-fill RBC allows the restoration to be performed much faster, thereby reducing the time of treatment, patient’s discomfort, and failure probability. Thus, the flowable bulk-fill RBC technique is more preferable for the pediatric dentist. On the contrary, possible concerns about the durability of structures fade into the background, since the maximum expected lifetime of the restoration is about 6 years, as the children usually start losing their primary teeth at around 6 years of age, with the average age for loss of primary molars around 10–12 years of age.

## 5. Conclusions

According to the results, both restorative techniques with both materials (*Ceram•X mono* and *SDR*) have provided almost identical results. With that, the easier and faster application technique of bulk-fill material (*SDR*) is extremely valuable for pediatric dentistry. Thus, one should consider the described technique as a treatment option and agree with Ehlers et al. (2013) that regarding the expected longevity of a primary tooth restoration, the presented fast-track approach seems to be an appropriate option.

## 6. Limitation of the Study

The results of the study may have insufficient evidence given the relatively small number of patients and the short observation period.

## Figures and Tables

**Table 1:** The modified Ryge criteria used in the study.

Characteristic	Rating	Criteria
Color match	Alpha	Restoration matches adjacent tooth structure in color and translucency
	Bravo	Mismatch in within an acceptable range of tooth color and translucency
	Charlie	Mismatch is outside the acceptable range
Cavosurface marginal discoloration	Alpha	Absence of marginal discoloration
	Bravo	Presence of marginal discoloration limited and not extended
	Charlie	Evident marginal discoloration penetrated toward the pulp chamber
Marginal integrity	Alpha	Closely adapted, no visible crevice
	Bravo	Visible crevice, explorer will penetrate
	Charlie	Crevice in which dentin is exposed
Surface texture	Alpha	Smooth surface
	Bravo	Slightly rough or pitted, can be refinished
	Charlie	Rough, cannot be refinished
Postoperative sensitivity	Alpha	Absence of dentinal hypersensitivity
	Bravo	Presence of dentinal hypersensitivity
Secondary caries	Alpha	No evidence of caries
	Bravo	Caries is evident
Fracture	Alpha	No evidence of fracture
	Bravo	Evidence of fracture

The Mann–Whitney U-test was used because the samples are not normal (Shapiro–Wilk test has failed, *p* < 0.050) and the sizes of the samples were different. The SigmaPlot 12.5 computer program was used. All data were expressed as the mean ± SD, with *p* < 0.05 considered statistically significant.

**Table 2:** The summary of the clinical findings of Ryge criteria at the end of 24 months.

Time	Color Match			Cavosurface Marginal Discoloration			Marginal Integrity			Surface Texture			Postoperative Sensitivity		Secondary Caries		Fracture	
Prevalence of Scores/Complications, %	A	B	C	A	B	C	A	B	C	A	B	C	A	B	A	B	A	B
*CeramX mono + Prime* and *Bond NT* (prevalence of scores/complications, %)																		
Baseline	100	0	0	100	0	0	100	100	0	100	0	0	100	0	100	0	100	0
6 months	100	0	0	100	0	0	100	100	0	100	0	0	100	0	100	0	100	0
18 months	100	0	0	92.6	7.4	0	100	100	0	96.3	3.7	0	100	0	100	0	100	0
24 months	100	0	0	92.6	7.4	0	96.3	3.7	0	96.3	3.7	0	100	0	100	0	100	0
*SDR + Prime* and *Bond NT* (prevalence of scores/complications, %)																		
Baseline	100	0	0	100	0	0	100	100	0	100	0	0	100	0	100	0	100	0
6 months	100	0	0	100	0	0	100	100	0	100	0	0	100	0	100	0	100	0
18 months	100	0	0	92.6	7.4	0	100	100	0	100	0	0	100	0	100	0	100	0
24 months	100	0	0	92.6	7.4	0	92.6	7.4	0	100	0	0	100	0	96.3	3.7	100	0

**Table 3:** The results of Mann–Whitney U-test.

Criterion	U	P
Color match	72.00	1.00
Cavosurface marginal discoloration	72.00	1.00
Marginal integrity	72.00	1.00
Surface texture	68.00	0.82
Postoperative sensitivity	6.00	0.69
Secondary caries	4.00	0.34
Fracture	4.00	0.34

## Supplementary Materials

The following are available online at https://www.mdpi.com/2304-6767/7/4/94/s1.
