# Supplementary material for: Flowable Bulk-Fill Materials Compared to Nano Ceramic Composites for Class I Cavities Restorations in Primary Molars: A Two-Year Prospective Case-Control Study
Source: Dent J (Basel). 2019 Sep 25;7(4):94. doi: 10.3390/dj7040094 (PMC6960826; doi:10.3390/dj7040094)
Supplement: Supplementary file 1 [file dentistry-07-00094-s001.zip › Supplement 1.pdf]

Приложение к медицинской карте №__________ от ____________________201_ г.

Информированное согласие на проведение медицинского вмешательства на лечение кариеса, глубокого кариеса. Основание Федеральный закон от 21.11.2011 № 323-ФЗ «Об основах охраны Здоровья граждан в Российской Федерации»)

Я, (Ф.И.О.) ____, обращаюсь в медицинскую фирму «Витал ЕВВ» для лечения заболевания (указать) ____

1. Я, получил(а) информацию об особенностях течения заболевания, вероятной длительности лечения, о методах лечения, примерной стоимости, о вероятном прогнозе заболевания. Мне предложен план обследования и лечения, даны полные разъяснения о характере, целях и продолжительности, о возможных неблагоприятных эффектах диагностических процедур, а также о том, что предстоит делать мне во время их проведения.

Мне разъяснено, что я должен(на) пройти обязательные диагностические исследования для назначения лечения, адекватного состоянию моего здоровья на настоящий момент. В процессе лечения могут проводиться дополнительные диагностические исследования, как для контроля лечения, так и для выявления другой патологии.

Мне объяснено, и я понял(а), что существует вероятность того, что во время осуществления основного плана лечения выяснится необходимость в частичном или полном изменении данного плана лечения, включая дополнительные врачебные процедуры, которые невозможно достоверно и в полной мере предвидеть заранее.

2. **Целью лечения кариеса** является устранение его проявлений для предотвращения развития осложнений (пульпит, периодонтит). Кариес - это разрушение твердых тканей зуба, которое начинается с деминерализации (растворения минеральных веществ, входящих в состав зуба), с последующим образованием полости. Данный процесс происходит под действием бактерий, которые ферментируют углеводистые соединения, образуя при этом кислоты. Под действием данных кислот происходит выход минеральных компонентов из твердых тканей зуба. Факторам способствующими развитию кариеса является неудовлетворительная гигиена полости рта, диета, богатая легко ферментируемыми углеводами, наследственность и прочее. Кариес протекает в несколько этапов: пятно (белое или темное), поверхностный кариес, средний кариес, глубокий кариес.

3.**Сущность медицинского вмешательства.** Лечение кариеса заключается в удалении пораженных тканей зуба с использованием механической и хирургической (в некоторых случаях ультразвуковой и/или лазерной) обработки в пределах здоровых тканей зуба с последующим восстановлением формы зуба прямым или непрямым способом. При прямом способе восстановление пломбировочный материал вносится в полость зуба, при непрямом способе (как правило, при больших по размеру кариозных полостях) в лаборатории изготавливается вкладка и фиксируется на цемент.

4**. Альтернативные методы лечения**. Методов лечения кариеса без механической и медикаментозной обработки кариозной поверхности не существует.

5. Риск при проведении лечения и возможные последствия:

1. Размеры кариозной полости (в том числе и под старой пломбой) невозможно точно определить до ее раскрытия. Соответственно, говорить точно об объёмах и методах восстановления разрушенного зуба (пломба, вкладка, коронка) можно будет только после полного удаления кариозных тканей. В некоторых случаях зубы, имеющие значительные поражения, не могут быть восстановлены и подлежат удалению.

2. При восстановлении эстетических показателей врачей руководствуется классическими параметрами формы зубов, но часто объективные клинические данные пациента (цвет и объём оставшейся зубной ткани реставрируемого зуба, цвет и форма рядом стоящих зубов, состояние прикуса и т.д.), накладывают ограничения на достижение эстетического результата, ожидаемого пациентом.

3. После постановки пломбы в ходе лечения кариеса возможен временный дискомфорт в зубе (постоперативная чувствительность). В редких случаях сохранение болезненности может быть до 6 месяцев.

4. При постановке пломбы в случае глубокого кариеса, в течение нескольких дней может появиться боль в зубе, если в пульпе уже есть необратимое воспаление, протекавшее до лечения бессимптомно. Это связано с тем, что дентин способен пропускать часть бактерий через свою толщу в пульпу зуба, поэтому при повреждении в пульпе формируется очаг постоянного (хронического) воспаления, выраженность которого зависит от протяженности кариозного дефекта. Очаг воспаления сохраняется после удаления кариеса и пломбировании зуба, а его ликвидация зависит от состояния иммунной системы. По этой причине, спустя некоторое время после пломбирования зуба по поводу неосложненного кариеса, может потребоваться эндодонтическое вмешательство (лечение корневых каналов). Обострение заболевания в пульпе может возникнуть и в более отдалённые сроки, и это тоже приведет к эндодонтическому лечению.

5. Кроме того, даже при соблюдении наивысшей степени заботливости и осмотрительности, какая требуется по характеру выполняемой технологии от врача, возможны следующие риски:

- при механической обработки (препарировании) зуба может произойти вскрытие полости зуба вследствие анатомических особенностей строения зуба;
- возможно развитие медикаментозного пульпита (периодонтита) вследствие индивидуальной реакции на применяемые медикаменты;
- перелом зуба;

- увеличение количества зубного налета в области границы ткани зуба и пломбы вследствие невозможности достижения идеального перехода;
Любые осложнения могут привести к удалению зуба, возникновения очага инфекции в челюстно-лицевой области, длительному болевому синдрому.

Мною дано согласие на осуществление местной анестезии, мне ясны и понятны возможные риски и осложнения, связанные с местной анестезией: поломка иглы, боль и жжение при инъекции, длительная или постоянная парестезия, тризм, гематома, инфекция, отёк, повреждение и некроз тканей, парез лицевого нерва, системные, токсические, аллергические реакции (вплоть до анафилактического шока), травмирование мягких тканей щеки, языка, губы в результате накусывания во время действия анестезии при несоблюдении рекомендаций лечащего врача.

Выбор анестезиологического пособия я доверяю своему лечащему врачу, информирующему меня о последствиях и нежелательных результатах анестезии.

Я извещен(а) о необходимости соблюдения мной режима в ходе лечения, регулярно принимать назначенные препараты, немедленно сообщать врачу о любом ухудшении моего самочувствия, согласовывать с врачом прием любых не прописанных препаратов, например, для лечения простуды, гриппа, головной боли и т.п.).

Я подтверждаю, что предложенная мне анкета о перенесённых мною заболеваниях и имевших место осложнениях заполнена мною лично и содержащаяся в ней информация достоверна.

Мне известно, что медицинская услуга относится к категории опасных, и возможные осложнения в процессе лечения зависят не только от медицинского вмешательства, но и от состояния моего организма.

Я получил (а) полную информацию о гарантийных сроках, действующих в стоматологической клинике "ВиталЕВВ": на корневую пломбу – 3 года; на лечение кариеса -1-3 года в зависимости от гигиенических индексов и общего состояния здоровья, клинической ситуации.
Я ознакомлен (а) со всеми требованиями, которые обязываюсь соблюдать. Я понимаю, что в случае их несоблюдения я лишаюсь права на гарантию.
Понимая сложность предстоящего лечения, я обязуюсь приходить на контрольные осмотры два раза в год и следить за гигиеной полости рта. Я имел (а) возможность задать все интересующие меня вопросы.
Я ознакомился (ась) с данным Приложением и понимаю, что последнее влечет для меня правовые последствия.
Подпись пациента ____________________________________ Дата ______________________________.
Подпись врача ____
Примечание: при приеме детей до 18 лет обязательно подписывается родителем

ДАТА	№ ЗУБА	ПОДПИСЬ ПАЦИЕНТА	ПОДПИСЬ ВРАЧА

